# Oil Price Uncertainty, Transport Fuel Demand and Public Health

**DOI:** 10.3390/ijerph14030245

**Published:** 2017-03-01

**Authors:** Ling-Yun He, Sheng Yang, Dongfeng Chang

**Affiliations:** 1 School of Economics, Jinan University, Guangzhou 510632, China; 2 Institute of Resource, Environment and Sustainable Development Research, Jinan University, Guangzhou 510632, China; 3 School of Economics and Management, Nanjing University of Information Science and Technology, Nanjing 210044, China; 4 Huaxia Bank and Renmin University of China Joint Postdoctoral Research Station, Beijing 100005, China; yangsheng11111@163.com; 5 School of Economics, Shandong University, Jinan 250100, China

**Keywords:** road transport, air pollution emissions, oil prices, public health, fuel demand price elasticities, pollution emission elasticities

## Abstract

Based on the panel data of 306 cities in China from 2002 to 2012, this paper investigates China’s road transport fuel (i.e., gasoline and diesel) demand system by using the Almost Ideal Demand System (AIDS) and the Quadratic AIDS (QUAIDS) models. The results indicate that own-price elasticities for different vehicle categories range from −1.215 to −0.459 (by AIDS) and from −1.399 to −0.369 (by QUAIDS). Then, this study estimates the air pollution emissions (CO, ${\mathrm{NO}}_{x}$ and ${\mathrm{PM}}_{2.5}$) and public health damages from the road transport sector under different oil price shocks. Compared to the base year 2012, results show that a fuel price rise of 30% can avoid 1,147,270 tonnes of pollution emissions; besides, premature deaths and economic losses decrease by 16,149 cases and 13,817.953 million RMB yuan respectively; while based on the non-linear health effect model, the premature deaths and total economic losses decrease by 15,534 and 13,291.4 million RMB yuan respectively. Our study combines the fuel demand and health evaluation models and is the first attempt to address how oil price changes influence public health through the fuel demand system in China. Given its serious air pollution emission and substantial health damages, this paper provides important insights for policy makers in terms of persistent increasing in fuel consumption and the associated health and economic losses.

## 1. Introduction

**Figure 1:**
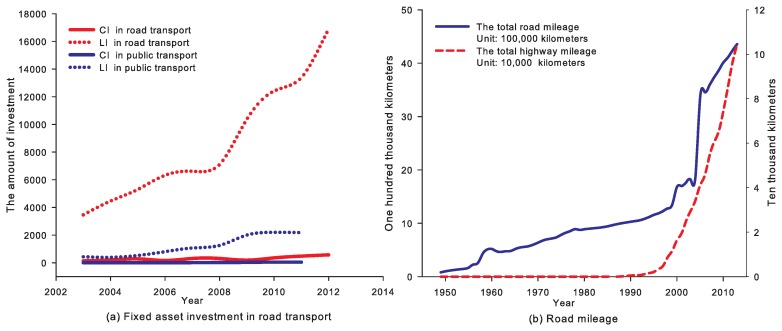
Fixed asset investment in road transport and road mileage in China (CI and LI stand for investment from the central government and local governments respectively; data source: NBSC, http://data.stats.gov.cn/index).

**Figure 2:**
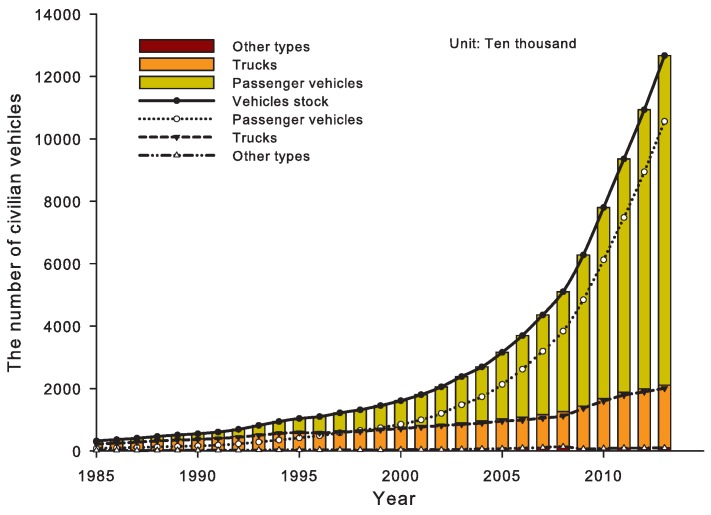
The civil vehicles in China (source: NBSC, http://data.stats.gov.cn/index).

**Figure 3:**
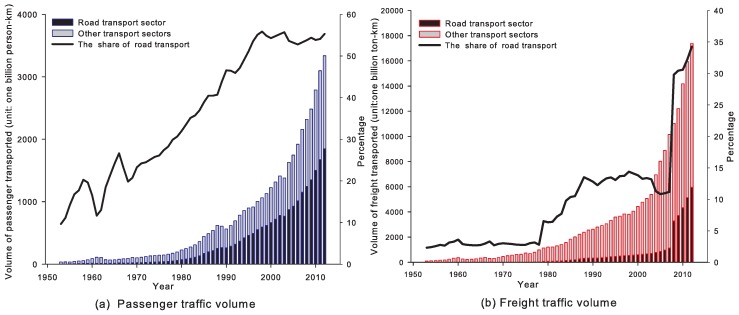
The freight and passenger traffic volumes in China (data source: NBSC, http://data.stats.gov.cn/index).

**Figure 4:**
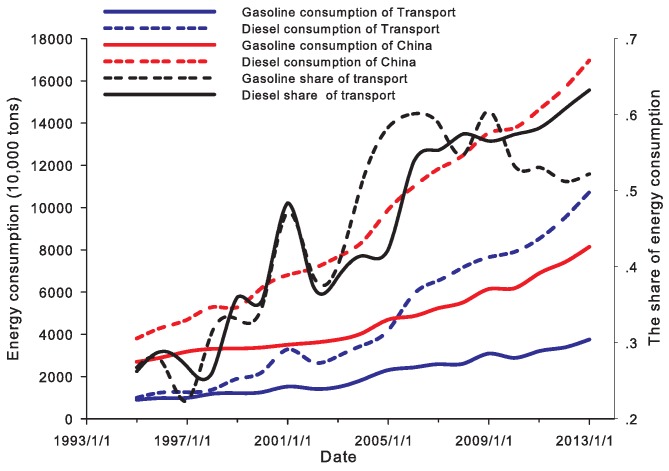
The gasoline and diesel consumption in China (source: National Bureau of Statistics of China (NBSC), http://www.stats.gov.cn).

In recent years, China’s rapid economic growth has correlated with huge energy consumption, especially for the road transport sector (for example, in recent years, China’s road transport sector has had enormous growth in the road transport infrastructure investment (see Figure 1), road (and highway) mileage (see Figure 1), the stock of vehicles (see Figure 2), freight and passenger traffic volumes (see Figure 3), etc.). In 2012, China’s total consumption of gasoline and diesel reached 81,410,000 tonnes and 169,660,000 tonnes respectively. The gasoline and diesel consumption of road transport sector was 9,000,000 tonnes and 9,980,000 tonnes in 1994; while, in 2012, they increased to 37,530,000 tonnes and 107,270,000 tonnes respectively, which accounted for 46% and 63% in the road transport sector in 2012 (see Figure 4).

As a consequence, China’s rapid economic growth and energy consumption resulted in serious environmental issues, especially air pollution emissions, which caused substantial losses to economic development and public health [1,2,3,4,5]. It has been shown that about two-thirds of China’s cities have not attained the ambient air quality standards applicable to urban residential areas [1]. According to The World Bank [6], the economic burden of premature mortality and morbidity associated with air pollution in China accounted for 157.3 billion RMB yuan (1.16% of GDP) in 2003. In addition, Chen et al. estimate that total suspended particles (TSPs), one of the major air pollution emissions, is causing the 500 million residents in Northern China to lose more than 2.5 billion life years of life expectancy [7].

Due to the large-scale investment driven by huge transport demand (see Figure 1), the civil vehicle stock, passenger and freight volumes have been increasing drastically during the past three decades (see Figure 2 and Figure 3).

As a result, the road transport sector is found to be one of the major emitters and responsible for serious air pollution and huge public health losses in China [4,8,9,10,11], especially in urban areas [1,12,13,14]. In 2012, the total vehicle emissions reached to 46.12 million tonnes. Specifically, the emissions of ${\mathrm{NO}}_{x}$, ${\mathrm{PM}}_{2.5}$, HC (hydrocarbon) and CO were 6.4, 0.622, 4.382 and 34.71 million tonnes, respectively (source: China Vehicle Emission Control Annual Report 2013). For urban areas such as Beijing, Has and Wang find that 74% of the ground ${\mathrm{NO}}_{x}$ in Beijing was attributed to vehicles, while only 2% and 13% were emitted by power plants and industry respectively [1]. They also find that vehicle exhaust accounted for 46% of the total VOCs emission in Beijing. In addition, Guo et al. [13] estimate that the total economic costs of health impacts due to air pollution contributed from transport in Beijing during 2004 to 2008 was 272, 297, 310, 323, 298 million U.S. dollars (mean values), respectively, which accounted for 0.52%, 0.57%, 0.60%, 0.62% and 0.58% of annual local GDP.

**Figure 5:**
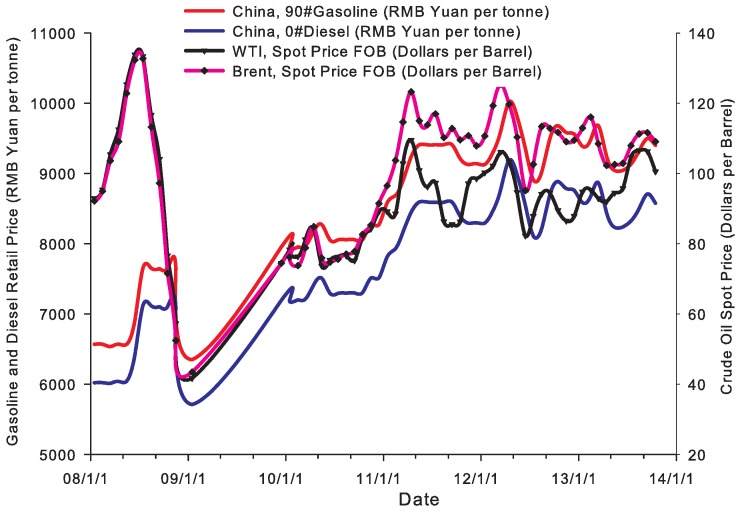
China’s gasoline and diesel retail prices and international crude oil spot price (source: National Bureau of Statistics of China (NBSC) and U.S. Energy Information Administration (EIA).

After the 2008 financial crisis, crude oil prices experienced drastic fluctuations. For example, from 2008 to 2009, the annual average crude oil prices of WTI (West Texas Intermediate) and Brent fell by 38% and 36%, respectively. In 2010, crude oil prices of WTI and Brent increased 28% and 29% than those in 2009, respectively. However, WTI crude oil price in 2013 was only 4% higher than that in 2012; in contrast, Brent crude oil price decreased by 3% (source: U.S. Energy Information Administration (EIA), http://www.eia.gov; we can also see this trend from Figure 5). Similarly, since 2008, China’s gasoline and diesel prices, which are based on international crude oil price, also experienced sharp volatility (see Figure 5) (according to the “Oil price management (tentative) of China”, the gasoline and diesel retail prices are usually determined by the following mechanism in China. The National Development and Reform Commission (NDRC) sets the gasoline and diesel highest retail prices, which are mainly determined on the basis of the international crude oil price, for provinces (autonomous regions and municipalities) or central cities; therefore, the international crude oil price is the benchmark for China’s oil price, and China is a price taker rather than a price maker). From August 2014, as international crude oil price fell sharply from $100 per barrel to around $50 per barrel in February 2015, nearly a 50 percent decline (source: U.S. Energy Information Administration (EIA), http://www.eia.gov), China’s gasoline and diesel retail prices appeared as a rare “thirteen losing streak”.

The role of oil prices in the macroeconomy has been the focus of a large number of economic studies [15,16,17,18,19,20,21]. One important parameter for determining the consequences of crude oil price shocks for the macroeconomy is the price elasticity of the demand for gasoline [22]. As shown in Table 1, the demand for gasoline has been investigated extensively using different methodologies.

In recent years, more studies have used flexible demand system methods to estimate the gasoline demand. For example, the normalized quadratic (NQ) method is applied to examine inter-fuel substitution elasticities at the sector and aggregate levels [23,24]. By employing Canadian household survey data and using three of the flexible functional forms (the Almost Ideal Demand System (AIDS), the Quadratic AIDS (QUAIDS) and the Minflex Laurent model), Chang and Serletis estimate that the own-price elasticity for gasoline demand in the transportation sector is between −0.738 and −0.570 [22].

On the one hand, as mentioned earlier, China’s rapid economic growth and huge energy consumption resulted in serious air pollution emissions. Especially, the road transport sector has been blamed as one of the major emitters, which caused huge losses to public health in China. On the other hand, after the 2008 financial crisis, oil prices experienced more frequent and dramatic fluctuations. Therefore, there are some extremely important issues to be investigated: What effects do oil price changes have on the road transport sector in China? Furthermore, how do oil prices affect the road transport pollution emissions and public health? Unfortunately, by now, there are still no clear and satisfactory solutions to these issues.

In this paper, we use the road transport panel data from the majority of prefecture-level cities in China and estimate the road transport fuel (i.e., gasoline and diesel) demand through two flexible demand systems (the AIDS and the QUAIDS models). After that, we investigate the impact of pollution emissions and public health from oil price shocks by means of pollution emission elasticities, as well as air quality and health effects evaluation models. Our framework, to the best of our knowledge, is the first attempt to study the transmission mechanisms between the oil price changes and public health effects in China.

The rest of the paper is organized as follows. Section 2 provides a discussion of the framework of this study; Section 3 discusses the data and some key parameters; Section 4 presents the empirical results for road transport fuel demand system and health effects; Section 5 concludes the paper.

## 2. The Modeling Framework

### 2.1. The Price and Expenditure Elasticities of Gasoline and Diesel Demand

The first step of this study is to estimate the price and expenditure elasticities of fuel (gasoline or diesel) demand for all vehicle types using the AIDS model [32] and the QUAIDS model [33]. In this paper, we assume that the vehicles’ fuel (gasoline or diesel) demands are weakly separable from other vehicle types and other economic sectors’ fuel demands. In reality, fuel price, regional, economic growth and other factors may have an impact on vehicle mileage traveled (VMT) for the various vehicle types. However, considering the frequency and magnitude of changes in oil price, oil price is still the most important factor in vehicle energy consumption. Therefore, this paper does not consider the possibility of error terms simultaneity or endogeneity bias caused by some unobservable aspects such as provinces or years. In addition, this paper focuses on the fuel consumption of motor vehicles in Chinese cities, rather than individual consumers. In addition, as we know, there is no national data about the user cost of operating the various types of automobiles (such as depreciation, etc.). Therefore, given the current limitations and difficulties in the field of research, we do not consider these factors, such as the prices and the user cost of the vehicles, etc.

The AIDS model has the following demand functions in budget share form:

(1) $$w_{i}=\alpha_{i}+\sum_{j=1}^{n}\gamma_{ij}\ln p_{j}+\beta_{i}\ln\left(\frac{y}{P}\right)+\nu_{i}$$

(2) $$\ln P=\alpha_{0}+\sum_{j=1}^{n}\alpha_{j}\ln p_{j}+\frac{1}{2}\sum_{i=1}^{n}\sum_{j=1}^{n}\gamma_{ij}\ln p_{i}\ln p_{j}$$

where both *i* and *j* indicate vehicle types. When vehicle type *i* (or *j*) is determined, the fuel type it uses is also determined. *n* stands for the number of vehicle types (*n* = 10). $w_{i}$ represents the vehicle fuel expenditure share of vehicle type *i*. $p_{i}$ (or $p_{j}$) means the fuel price of the *i*-th (or *j*-th) type of vehicle; while $p_{i}$ = $p_{j}$ implies that the *i*-th and *j*-th vehicles employ the same kind of fuel; otherwise ($p_{i}$ ≠ $p_{j}$), they use different types of fuel (This study includes a total of ten vehicle types using gasoline or diesel as fuel. For more information in detail, see the Section 3). *y* represents total expenditure on fuel in the demand system. *P* indicates the translog price index. $\gamma_{ij}$ and $\beta_{i}$ are parameters to be estimated; $\nu_{i}$ is the error term.

According to [33], the QUAIDS share equations are as follows:

(3) $$w_{i}=\alpha_{i}+\sum_{j=1}^{n}\gamma_{ij}\ln p_{j}+\beta_{i}\ln\left[\frac{y}{a(p)}\right]+\frac{\lambda_{i}}{b(p)}{\left\{\ln\left[\frac{y}{a(p)}\right]\right\}}^{2}$$

(4) $$\ln a\left(p\right)=\alpha_{0}+\sum_{i=1}^{n}\alpha_{i}\ln p_{i}+\frac{1}{2}\sum_{i=1}^{n}\sum_{j=1}^{n}\gamma_{ij}\ln p_{i}\ln p_{j}$$

(5) $$b\left(p\right)=\prod_{i=1}^{n}{\left(p_{i}\right)}^{\beta_{i}}$$

(6) $$\lambda \left(p\right)=\sum_{i=1}^{n}\lambda_{i}\ln p_{i}$$

where $w_{i}$ is the *i*-th budget share; $\sum_{i=1}^{n}\lambda_{i}=0$, and i=1,…,n denotes the number of vehicle types. *α*, *β*, *γ* and *λ* are parameters. By setting $\lambda_{i}=0$ (i=1,…,n), Equation (3) reduces to the AIDS share equation.

For the AIDS and the QUAIDS models, economic theory imposes several restrictions on the parameters of the models:

- the Slutsky symmetry condition given by $\gamma_{ij}$ = $\gamma_{ji}$ for any *i* and *j*;
- the homogeneity condition given by $\sum_{j=1}^{n}\gamma_{ij}=0$ for any *j*;
- the adding-up restriction imposed as $\sum_{i=1}^{n}w_{i}=1$ or, equivalently, $\sum_{i=1}^{n}\alpha_{i}=1$, $\sum_{i=1}^{n}\beta_{i}=0$ and $\sum_{i=1}^{n}\gamma_{ij}=0$.

In addition, the price elasticity of fuel demand can be calculated by:

(7) $$e_{ij}=-\delta_{ij}+\frac{p_{j}}{w_{i}}\frac{\partial w_{i}}{\partial p_{j}}$$

for i,j=1,…,n, where $\delta_{ij}$ is the Kronecker delta (i.e., $\delta_{ij}$ = 1 when i=j and zero otherwise).

The fuel expenditure elasticity is given by:

(8) $$e_{i}=1+\frac{y}{w_{i}}\frac{\partial w_{i}}{\partial y}$$

### 2.2. The Air Pollution Emission Elasticities

The second step is to investigate the changes of air pollutants caused by fuel demand changes. Inspired by the concept of nutrient elasticities applied in studies on human nutritional intake [34,35], we propose the concept of air pollution emission elasticities, which reflect how the changes in different types of vehicle activities impact the air pollution emissions. Specifically, in this study, the air pollution emissions refer to the emissions of three major pollutants from various vehicle types, including CO, ${\mathrm{NO}}_{x}$ and ${\mathrm{PM}}_{2.5}$ (For example, through the nutrient elasticities, Zheng and Henneberry [35] study the nutrient demand of three major nutrient substances (i.e., calorie, protein and fat), which were obtained from the foods (such as grain, oil, meat, eggs, etc.) consumed by a household. In this paper, for the air pollution emission elasticities, the three kinds of pollutants (i.e., CO, ${\mathrm{NO}}_{x}$ and ${\mathrm{PM}}_{2.5}$) are similar to the three nutrients, and the motor vehicles are similar to the foods.).

Based on the demand elasticities computed in the first step, the air pollution emissions’ elasticities are given by:

(9) $$\pi_{ki}=\sum_{i=1}^{N}e_{ij}a_{ki}q_{i}/\psi_{k}$$

(10) $$\eta_{k}=\sum_{i=1}^{N}e_{i}a_{ki}q_{i}/\psi_{k}$$

(11) $$\psi_{k}=\sum_{i=1}^{N}a_{ki}q_{i}$$

where $\pi_{ki}$ denotes the price elasticity of air pollution emissions, which illustrates the weighted average of all own- and across-price emission elasticities for air pollutant *k* in response to the price of fuel for the *i*-th vehicle type; $\eta_{k}$ represents the expenditure elasticity of air pollutant emissions, which indicates the weighted average of all air pollutant *k* emission elasticities with respect to expenditures; $q_{i}$ denotes the quantity of fuel used by vehicle type *i*; $\psi_{k}$ stands for the total amount of pollutant emissions from all types of vehicles; and $a_{ki}$ is the pollution emission factor, which means that the pollutant *k* emission the from unit mileage of the *i*-th vehicle type.

### 2.3. A Simplified Air Quality Health Effects Estimation

Through the previous two steps, we estimated the impact of oil prices on fuel demand (by the fuel demand elasticities) and pollution emissions (air pollution emission elasticities) of road transport. After obtaining the fuel demand elasticities and pollution emission elasticities of road transport, the third step is to estimate the changes in air pollutant concentrations caused by road transport emissions and then to evaluate the corresponding public health damage.

In this study, we use the air concentration model to estimate the changes in air quality. To simplify the question, we follow Chen and He’s approach [10] and employ a fixed box model whereby China is represented by a parallelepiped with the uniform pollutant dispersion:

(12) $$C_{k}=b_{k}+\frac{S_{k}L}{Hu}$$

where $C_{k}$ stands for the concentration of pollutant *k* in the entire site (μg/m${}^{3}$); $b_{k}$ the background concentration of pollutant *k* under natural conditions (μg/m${}^{3}$); $S_{k}$ the emission rate of pollutant *k* (μg/s/m${}^{2}$); *L* the length (m), *H* the mixing height (m) and *u* the wind velocity (m/s).

Given the difficulty of obtaining the meteorological parameters ($S_{k}$, *L*, *H*, *u*), specifically in China at the national level, we assume these parameters ($b_{k}$, *L*, *H*, *u*) are constant under the baseline situation ($S_{k1}$, $C_{k1}$) and the future situation ($S_{k2}$, $C_{k2}$). Taking into account the time span of the year, it is reasonable to assume that pollutants are uniformly mixed in the volume (at the national level). Moreover, by using this typical box model, these above variables (such as the length, mixing height and wind velocity) are assumed to be constant in the basic and future scenarios, which simplify Model (12) to Model (13). Under this assumption, we simplify the model and estimate the change of air pollutant *k* by:

(13) $$\frac{S_{k2}}{S_{k1}}=\frac{C_{k2}-b_{k}}{C_{k1}-b_{k}}=\frac{E_{k2}}{E_{k1}}$$

where $S_{k1}$ and $S_{k2}$ are the baseline emission rate and the future emission rate of pollutant *k* respectively. $C_{k1}$ and $C_{k2}$ are the annual average baseline and the future concentration levels, respectively.

We further assume that $E_{k1}$ and $E_{k2}$ are the baseline emissions and future emissions of pollutant *k* from road transport sector in China, respectively. Thus, the ratio of $E_{k2}$ to $E_{k1}$ reflects the change of the pollutant *k* emissions from the road transport sector. Under the assumption of the uniform emission rate, it is reasonable to think of the ratio of $S_{k2}$ to $S_{k1}$ as the ratio of the $E_{k2}$ to $E_{k1}$. Considering data reliability, the Chinese vehicle pollution emissions data in 2012 were chosen as baseline emissions $E_{k1}$ (see Table 2).

Once the change of air pollutant concentration level is determined, the consequent risks in health outcomes can be assessed using exposure-response (ER) functions from epidemiological studies, which typically include mortality (acute and chronic), respiratory hospital admission, cardiovascular hospital admission, restricted activity day, work loss day, asthma attack, etc. [10]. Due to the data availability, the quantifiable health effects in our analysis only include acute mortality. As a consequence, please note that the real health losses might be significantly underestimated in this study. Even so, the estimated results are formidably substantial (please see Table 1). The number of premature deaths from acute exposure [10] is estimated by:

(14) $$Case^{AM}=\sum_{k}ER_{k}^{AM}\times C_{k}\times N\times M$$

where $ER_{k}^{AM}$ represents the ER coefficients for mortality from acute exposure to the pollutant *k*. $C_{k}$ denotes the concentration level of pollutant *k* (see Equation (13)). *N* illustrates the total population exposed to the air pollution. *M* means the overall mortality rate.

Due to the limitations of linear health effects models, this paper also applies the nonlinear health effect model Equations (15)–(17) [38,39,40] to evaluate the health loss and acute mortality as follows:

(15) $$RR_{k}=e^{\beta_{k}\left(C_{k}-b_{k}\right)}$$

(16) $$AF_{k}=\frac{RR_{k}-1}{RR_{k}}$$

(17) $$E_{k}=AF_{k}\times N\times M$$

where $RR_{k}$ is the relative risk of pollutant *k* (i.e., CO, ${\mathrm{NO}}_{x}$ and ${\mathrm{PM}}_{2.5}$). $\beta_{k}$ is the ER coefficients for acute exposure of pollutant *k*; $C_{k}$ is the pollutant concentration for the case under study (mg/m${}^{3}$ for CO; μg/m${}^{3}$ for ${\mathrm{NO}}_{x}$ and ${\mathrm{PM}}_{2.5}$); and $b_{k}$ the background concentration of pollutant *k* under natural conditions (mg/m${}^{3}$ for CO; μg/m${}^{3}$ for ${\mathrm{NO}}_{x}$ and ${\mathrm{PM}}_{2.5}$); $AF_{k}$ is the attributable fraction of deaths to air pollutant *k*; $E_{k}$ is the total number of cases of premature mortality from acute exposure to the pollutant *k*.

For the public health loss assessment, we use the value of statistical life (VOSL) method, which represents an individual’s willingness to pay (WTP) for a marginal reduction in the risk of death [41]:

(18) $$VOSL=VOSL_{BL}\times {\left(\frac{I}{I_{BL}}\right)}^{e}$$

where VOSL and $VOSL_{BL}$ are the VOSL of the national level and the baseline level, respectively; while *I* and $I_{BL}$ represent the income per capita of the national and baseline levels, respectively. *e* is the elastic coefficient of WTP and is assumed to be one. Considering data reliability, the baseline $VOSL_{BL}$ is obtained from [41], which was $79,839 in Chongqing, one of the four province-level municipalities in China, in 2004.

## 3. Data and Key Parameters Description

In this paper, we investigate 306 cities in China over the period of 2002 to 2012. The data used in this paper include vehicle population, fuel (gasoline and diesel) prices, total population and overall mortality rate of China, air concentrations and exposure-response (ER) coefficients, annual average vehicle mileage traveled (VMT), fuel economy and pollution emission factors of different vehicles types, etc. (see Table 2 and Table 3 for a summary). Given that much of the data is not publicly available, a great effort has been made here in terms of the data collection and process.

Firstly, we use vehicle population data, which are specifically divided into ten vehicle categories (this paper uses the classification system of the Ministry of Public Security, which is used both for official statistical reporting by the National Bureau of Statistics of China (NBSC) [4,27]) (see Table 3). The panel dataset covers approximately 33,660 data from 306 cities in 25 provinces of mainland China from 2002 to 2012 (Because of data limitations, we are unable to obtain the data from the following areas: Xinjiang, Qinghai, Hong Kong, Macau and Taiwan. Moreover, we exclude municipalities’ data, namely Beijing, Tianjin, Shanghai and Chongqing. Data source: the NBSC, http://data.stats.gov.cn/index).

Secondly, we use the annual average vehicle mileage traveled (VMT) data in China. There have been some estimates on China’s VMT data (for example, see [51,54]). In addition, the VMT is related to the economic development level and road traffic infrastructure [54]). (In addition, the VMT is also affected by some other factors, such as vehicle age [51,54]. Due to data availability limitations, these factors are not considered in this study.). It is widely known that there are huge differences between different regions (cities) of China in geographic conditions, economic development and transportation infrastructure, etc. However, China’s existing VMT estimates in different cities are not readily available or substantially insufficient. Therefore, we use the national average VMT data in 2002 as the baseline data. Then, we estimate the provincial annual average VMT data for each type of vehicle from 2002 to 2012 by using the provincial average passenger data and freight transport distance data. Specifically, we calculate the ratio of provincial average passenger data to national average passenger data from 2002 to 2012. According to the ratio and national average VMT baseline data, we can further estimate the provincial VMT data for each type of passenger vehicle (i.e., large passenger vehicles (LPV)-D, medium PV (MPV)-G, small PV (SPV)-G, mini PV (MNPV)-G, public bus (PB)-D and Taxi-G in this paper) in every year. Similarly, for all kinds of truck (i.e., heavy duty truck (HDT)-D, medium duty truck (MDT-D), light duty truck (LDT-D) and mini truck (MNT-G) in this study), we use the ratio of provincial freight transport distance data to national freight transport distance data. On this basis, we assume that all of the cities within the same provincial region have the same VMT data. Our VMT estimates are supported by many survey papers and empirical studies in the existing literature (please see Table 3), indicating that our estimated results are reasonably acceptable.

Thirdly, the gasoline and diesel annual average prices, from 2002 to 2012, are used in this paper (source: Wind database, http://www.wind.com.cn). Since the international oil prices are the benchmarks of China’s oil prices and fundamentally important in China’s gasoline and diesel pricing mechanisms, the road transport sector is merely a price taker rather than a price maker. In this paper, we use the provincial gasoline and diesel annual average retail prices because gasoline and diesel prices in different cities within the same province are basically the same.

Finally, in 2012, the number of China’s total population was 1,354,040,000, while the overall mortality rate was 0.715%. According to Zhang et al. [41], the Chinese national average VOSL, which was calculated after inflation and exchange rate adjustment, was 855,642.81 RMB yuan in 2012 (for the inflation rate and exchange rate of RMB yuan to USD, see the NBSC, http://data.stats.gov.cn/index).

## 4. Empirical Estimation

### 4.1. Fuel Demand Elasticities

In this study, we use both the AIDS and the QUAIDS models to estimate the price elasticities of fuel demand (own-price and expenditure elasticities) for different vehicle types, which indicate their fuel demand changes caused by the changes in fuel prices. All fuel demand elasticities are calculated on the basis of parameter estimates and sample means of explanatory variables. In addition, standard errors of these elasticities are approximated using the delta method. For each model, the fuel demand elasticities contain 110 estimates of own- and cross-price, as well as expenditure elasticities for ten vehicle categories across the entire sample (Due to the limited space in this paper, the cross-price elasticities estimates are not listed. The full report of cross-price elasticities is available upon request.).

The results for own-price and expenditure elasticities are reported in Table 4, which are estimated by the AIDS model and demonstrate that all own-price elasticities are negative and statistically significant, which range from −1.215 to −0.459. Expenditure elasticities are positive and statistically significant, which range from 0.286 to 1.239. The estimated own-price elasticities vary across different vehicle categories. Except MPV-G (−1.215), the own-price elasticities values (in absolute values) for all studied vehicle categories are less than one, implying that they are not sensitive to fuel price changes. Especially for PB-D, the value is just −0.459. As we know, local governments usually support their urban transit system with price subsidies (such as the Beijing prolonged transit subsidies and low fares). Therefore, changes in the price of diesel have limited impacts on the public bus system. Conversely, fuel (gasoline) demands for MPV-G, of which its own-price elasticity value (in absolute value) is greater than one, are price-sensitive. Furthermore, in spite of the fact that SPV-G, MDT-D, LDT-D and Taxi-G are not sensitive to fuel prices, their price elasticities (in absolute value) are very close to one, which are −0.940, −0.937, −0.940, as well as −0.918, respectively, and may show that gasoline (or diesel) prices still have some influence on their fuel demand.

Furthermore, estimates by the QUAIDS model (see Table 5) are similar to those by the AIDS model. It suggests that all own-price elasticities are statistically significantly negative and range from −1.399 to −0.369; while expenditure elasticities are statistically significantly positive and vary from 0.233 to 1.214. In particular, the own-price elasticity values of SPV-G and Taxi-G are −1.399 and −1.373 respectively. It suggests that gasoline demands for SPV-G and Taxi-G are statistically elastic, which is different from the results based on the AIDS model. Instead, the other vehicle types (i.e., LPV-D, MPV-G, MNPV-G, HDT-D, MDT-D, LDT-D, MNT-G and PB-D) are not sensitive to fuel price changes. Especially, the own-price elasticity (in absolute value) of PB-D (−0.369) is much smaller than the other ones.

In addition, as shown in Table 4 and Table 5, the estimated own-price elasticities for most vehicle types are larger than those reported in other studies (see Table 1). This may be explained by the fact that most of the existing studies estimate the national gasoline (or diesel) demand elasticity, which includes industrial sector, agricultural sector, etc. Given the importance of fuel to the nation’s economy, especially for China, the fuel demand elasticities tend to be smaller in the short term at the national level. Even for some studies that only estimate the transport sector fuel demand elasticity, different types of vehicles have not been studied separately.

### 4.2. Pollution Emissions Elasticities

Using the estimates of fuel demand elasticities and pollution emission factors (Table 3), we calculate and report the elasticities of air pollution emission based on Equations (9) and (10) in Table 4 and Table 5. The pollution emissions price elasticities measure changes of air pollution emissions (CO, ${\mathrm{NO}}_{x}$ and ${\mathrm{PM}}_{2.5}$) in response to variations in fuel price for the studied vehicles groups; while the pollution emissions expenditure elasticities reflect the combined effect of all vehicles expenditure elasticities in the fuel demand system. As this study focuses on the pollution and health impacts due to fuel price changes, we pay special attention on the pollution emission price elasticities.

Similar to the price and expenditure elasticities of fuel demand, estimates for price elasticities of air pollution emissions are all negative, while expenditure elasticities are positive. This suggests that, when fuel (gasoline and diesel) prices rise, the road transport sector will reduce fuel demand and consumption, which will lead to a reduction of pollution emissions. Conversely, with all vehicles’ fuel expenditure increasing, the pollution emissions will increase. Moreover, the price elasticities of pollution emissions (in absolute value) are much lower than the corresponding price elasticities of fuel demands reported in Table 4 and Table 5. A plausible explanation is that the emissions of a specific air pollutant might be jointly influenced by all types of vehicles.

Specifically, according to the AIDS model, the CO emission price elasticities of SPV-G, MNPV-G, HDT-D and LDT-D are −0.326, −0.116, −0.178 and −0.134 respectively, which are much higher than other vehicles (in absolute value). It suggests that CO emissions from these types of vehicles are more sensitive to gasoline (or diesel) prices than the other vehicles. Moreover, the ${\mathrm{NO}}_{x}$ emission price elasticities of HDT-D (−0.300), LDT-D (−0.169) and MDT-D (−0.149) are larger than that of other vehicle types. While, for ${\mathrm{PM}}_{2.5}$ emission price elasticities, the values of SPV-G (−0.420), HDT-D (−0.119) and LDT-D (−0.139) are greater.

While using the QUAIDS model, the CO emission price elasticities of SPVG (−0.385), HDT-D (−0.125) and LDT-D (−0.119) are larger than other vehicles (in absolute value). For the emission price elasticities of ${\mathrm{NO}}_{x}$ and ${\mathrm{PM}}_{2.5}$, results based on the two models are quite similar. Both models suggest that for CO and ${\mathrm{PM}}_{2.5}$ emissions, the emission price elasticities of SPV-G, HDT-D and LDT-D are relatively larger than those of other vehicle types. Especially, ${\mathrm{NO}}_{x}$ emission price elasticities of the diesel vehicles (i.e., HDT-D, LDT-D and MDT-D) are significantly greater than those of gasoline vehicles, which indicate that diesel price has a larger impact on ${\mathrm{NO}}_{x}$ emissions.

To some extent, these findings are consistent with the reality of China’s vehicle emissions. In China, ${\mathrm{NO}}_{x}$ and ${\mathrm{PM}}_{2.5}$ emissions from trucks (diesel vehicles) are generally higher than those from passenger cars (gasoline vehicles). Conversely, the CO emissions from passenger cars (gasoline vehicles) are more than that from trucks (diesel vehicles). Particularly, heavy trucks are the major contributor to ${\mathrm{NO}}_{x}$ (source: China Vehicle Emission Control Annual Report 2013). Furthermore, the emission price elasticities depend on the fuel demand price elasticities (Equation (9)) and the proportion of pollution emissions associated with vehicle population, VMT, emission factors, etc. (some other factors may indirectly affect vehicle emissions, such as, in particular, vehicle emissions-control technologies, emissions standards [55]). For instance, the SPV-G has the greatest vehicle population in China (see Figure 3). In addition, as shown in Table 3, HDT-D and public bus using diesel fuel (PB-D) both have larger pollution emission factors and VMT; while emission price elasticities of PB-D are much smaller than those of HDT-D due to the smaller demand price elasticity for diesel.

### 4.3. Air Pollution Emissions and Health Effects from Oil Price Shocks

In this section, the air pollution emissions and public health effects are estimated under different oil price shocks. Applying Equation (13) and the elasticity estimates of fuel demand and air pollution emissions, we estimate the changes of air pollution emissions and corresponding concentrations under different scenarios extracted from real-world oil price shocks (see Panel A of Table 6). Furthermore, we estimate the public health effects (in this study, the public health effects include the premature deaths cases caused by acute exposure and corresponding economic losses) caused by road transport pollution under different oil price shocks (see Panel B of Table 6) using Equations (14), (17) and (18) (in order to avoid the limitations of a single health effect model and obtain more robust and accurate health loss estimates, we also use the non-linear health effect models (i.e., Equations (15)–(17)) to evaluate the health losses and compare the results of different methods).

Taking into account oil price fluctuations in recent years, we designed four scenarios from the real-world oil price shocks. In Scenarios 1 and 2, the prices of gasoline and diesel simultaneously rose by 30% (the extreme positive price shock scenario) and 5% (the average positive price shock scenario), respectively. In Scenarios 3 and 4, the prices of gasoline and diesel simultaneously declined by 40% (the extreme negative price shock scenario) and 3% (the average negative price shock scenario), respectively (see Table 6).

Results show that, when gasoline and diesel prices rise (Scenarios 1 and 2), air pollution emissions (emissions quantities and concentrations) and public health economic losses caused by the road transport sector decline correspondingly. In contrast, when gasoline and diesel prices decline (Scenarios 3 and 4), air pollution emissions and corresponding economic losses rise. For example, based on the results from the AIDS model, comparing with the base year 2012, when gasoline and diesel prices rise 30% simultaneously (Scenario 1), emissions of CO, ${\mathrm{NO}}_{x}$ and ${\mathrm{PM}}_{2.5}$ from road transport decrease by 963,940, 172,000 and 11,330 tonnes, respectively. In addition, concentrations drop by 0.008 mg/m${}^{3}$, 0.994 μg/m${}^{3}$ and 0.160 μg/m${}^{3}$, respectively. Based on the linear health effect model (Equation (14)), reductions of premature deaths caused by acute exposure are 2984, 12,515 and 650 cases, and the corresponding economic losses decrease about 2553.567, 10,708.197 and 556.189 million RMB, respectively. Furthermore, the total reduction of air pollution emissions from road transport reached 1,147,270 tonnes. As a result, the total number of premature deaths decreases by 16,149, and economic losses are reduced 13,817.953 million RMB yuan caused by air pollutant emissions from the road transport sector; while according to the non-linear health effect models (see Equations (15)–(17)), reductions of premature deaths are 2952, 11,934 and 648 cases; and economic losses decrease about 2525.704, 10,210.823 and 554.874 million RMB; the total premature deaths and economic losses decreased by 15,534 cases and 13291.4 million RMB. In contrast, when gasoline and diesel prices decrease 40% simultaneously (Scenario 3), pollution emissions increase by 1,529,690 tonnes; premature death cases and corresponding economic losses rise by 21,532 cases and 18,423.937 million RMB yuan respectively using the linear health effect model; premature death cases and economic losses rise by 20,690 cases and 17,703.193 million RMB yuan respectively using the non-linear health effect models.

Moreover, similar results have been found using the QUAIDS model. For instance, in Scenario 1, fuel prices rise 30%, meaning that 902,810 tonnes of air pollution emissions are avoided. Meanwhile, the premature deaths and economic losses decrease by 11,604 cases and 9928.509 million RMB yuan respectively from the linear health effect models, as well as decrease by 11,174 cases and 9560.635 million RMB yuan respectively from the linear health effect models. See Table 6 for results under other scenarios. It is obvious that the results based on the two different health effect models are quite similar and consistent without significant differences, which in turn indicates that this paper is robust and reliable.

## 5. Conclusions

In recent years, China’s rapid economic growth and huge energy consumption resulted in serious air pollution emissions, which caused substantial losses to public health. In particular, the road transport sector has been blamed as one of the major emitters in China. After the 2008 financial crisis, frequent and dramatic fluctuations in oil prices have had some significant impacts on China’s road transport sector, which is as a price-taker. Therefore, it is extremely important to study the effects of oil price shocks on public health in China. In this study, we estimate China’s road transport fuel demand system by using the AIDS and the QUAIDS models and investigate the impacts of pollution emissions and public health losses from the road transport sector under four scenarios of different oil price shocks.

We find that all of the own-price elasticities of fuel demand are negative and statistically significant, and they vary across different vehicle categories, which range from −1.215 to −0.459 based on the AIDS model and from −1.399 to −0.369 based on the QUAIDS model. In addition, expenditure elasticities are positive and statistically significant, which range from 0.286 to 1.239 based on the AIDS model and from 0.233 to 1.214 based on the QUAIDS model. In particular, results estimated by the AIDS model show that except for medium passenger vehicles, the own-price elasticities for all vehicle categories are inelastic, which indicate that Chinese drivers are not sensitive to fuel price changes, especially for public buses with the own-price elasticity of −0.459. Furthermore, estimates by the QUAIDS model are similar to results from the AIDS model except that the own-price elasticities of small passenger vehicles (−1.399) and taxi (−1.373) are inelastic.

Furthermore, price elasticities of air pollution emissions are also all negative, while pollution emission expenditure elasticities are positive. Specifically, results from both the AIDS and the QUAIDS models suggest that, for CO and ${\mathrm{PM}}_{2.5}$ emissions, the emission price elasticities of small passenger vehicles, heavy duty trucks and light duty trucks are comparatively larger than those of other vehicles. ${\mathrm{NO}}_{x}$ emission price elasticities of diesel vehicles are significantly greater than those of gasoline vehicles, implying that ${\mathrm{NO}}_{x}$ emissions are more sensitive to diesel price than gasoline price.

Finally, this study estimates the air pollutant emissions and health losses under different oil price shocks. Our results show that, when the increase of gasoline and diesel prices reduces the road transport fuel demand, public health losses caused by road transport will decrease. In contrast, when gasoline and diesel prices decline, meaning an increase in road transport fuel demand, the corresponding health losses rise. For instance, based on the AIDS model and the linear health effect model, we find that when gasoline and diesel prices rise 30% simultaneously, the total reduction of air pollution emissions from road transport sector reaches 1,147,270 tonnes. The total number of premature deaths decreases by 16,149, and the total economic loss is reduced by 13,817.953 million RMB; while based on the non-linear health effect model, the premature deaths and total economic loss decrease by 15,534 and 13,291.4 million RMB yuan respectively. According to the results from the linear health effect model, when gasoline and diesel prices decrease 40% simultaneously, pollution emissions increase by 1,529,690 tonnes; the total number of premature deaths and corresponding economic loss rise by 21,532 cases and 18,423.937 million RMB yuan respectively; while based on the non-linear health effect model, the premature deaths and economic loss rise by 20,690 cases and 17,703.193 million RMB yuan respectively. Furthermore, applying the QUAIDS model, we find that a 30% increase in fuel price will reduce air pollution emissions, premature deaths and economic losses in China by 902,810 tonnes, 11,604 cases (the linear health effect model) and 9928.509 million RMB yuan (the linear health effect model) respectively.

This paper is the first study that proposes a transmission mechanism between fuel demand and health damages in China using pollution emission elasticities. It investigates the impact of increasing demand for fuel from the road transport sector on the Chinese health and economic losses under four scenarios of oil price shocks. According to the Energy Information Administration, China is the world’s largest energy consumer and became the largest net importer of petroleum since 2014. Given its serious air pollution emission and substantial health damages, this paper provides important insights for policy makers in terms of persistent increasing in fuel consumption and the associated health and economic losses.

## Figures and Tables

**Table 1:** Some price elasticities of gasoline demand from earlier studies. AIDS, Almost Ideal Demand System; QUAIDS, Quadratic AIDS.

References	Research Methods	Price Elasticity Value
[25]	Econometric modeling analysis	Short-run: −0.26; long-run: −0.86
[26]	Meta-analysis of literature	Short-run: −0.26; long-run: −0.58
[27]	Regressions	Households in USA, short-run: −0.23
[28]	Literature survey	OECD countries: short-run: (−0.5,−0.34); long-run:(−1.35,−0.23)
[29]	AIDS	Households in USA: (−0.74,−0.18)
[30]	Regressions	China: short-run: −0.19; long-run: −0.56
[31]	Regressions	Transport sector in China: −0.269
[22]	AIDS, QUAIDS, Minflex Laurent	Households in Canada: (−0.738, −0.570)

Notes: (−0.5,−0.34) shows that the short-run price elasticity estimates range from −0.5 to −0.34. (−1.35,−0.23) shows that the long-run price elasticity estimates range from −1.35 to −0.23.

**Table 2:** Summary of the parameters of emissions, air concentrations and exposure-response (ER) coefficients ${}^{a}$.

Air Pollutants	Background Concentrations ${}^{b}$	ER Coefficients ${}^{c}$	Baseline Concentration ${}^{d}$	Baseline Emissions ^e^
CO	1	3.7	1.3	34.71
${\mathrm{NO}}_{x}$	10	0.13	47	6.4
${\mathrm{PM}}_{2.5}$	39	0.042	44.7	0.404

Notes: ${}^{a}$ We assume that all of the parameters are China’s national level value in 2012 due to limited data availability. ${}^{b}$ For the background concentration of CO, see [36]. For the background concentrations of ${\mathrm{NO}}_{x}$ and ${\mathrm{PM}}_{2.5}$, see [10], respectively. Unit: mg/m${}^{3}$ (CO), μg/m${}^{3}$ (${\mathrm{NO}}_{x}$, ${\mathrm{PM}}_{2.5}$). ${}^{c}$ The ER coefficients for acute exposure are expressed in mortality percent change per μg/m${}^{3}$ (mg/m${}^{3}$) change of pollutant concentration. For the ER coefficients of CO and ${\mathrm{NO}}_{x}$, please see [37]; and for the ${\mathrm{PM}}_{2.5}$, please see [10]. ${}^{d}$ For the baseline concentration of CO and ${\mathrm{NO}}_{x}$, see [37]. For the baseline concentration of ${\mathrm{PM}}_{2.5}$, see [10]. Unit: mg/m${}^{3}$ (CO), μg/m${}^{3}$ (${\mathrm{NO}}_{x}$, ${\mathrm{PM}}_{2.5}$). ^e^ In 2012, the ${\mathrm{PM}}_{10}$ emissions from road transportation in China were 0.622 million tons (data source: China Vehicle Emission Control Annual Report 2013.). We use 0.65 as the ${\mathrm{PM}}_{10}$ to ${\mathrm{PM}}_{2.5}$ conversion factor [10]. Therefore, the ${\mathrm{PM}}_{2.5}$ emissions from road transportation were 0.404 million tons. Unit: million tons.

**Table 3:** The annual average vehicle mileage traveled (VMT), fuel economy and pollution emission factors ${}^{a}$.

Vehicle Types ${}^{b}$	Baseline VMT ${}^{c}$ (1000 km)	Fuel Economy ${}^{d}$ (L/100 km)	Pollution Emission Factors (g/km)		
			CO ${}^{e}$	${\mathrm{NO}}_{x}$ ${}^{f}$	${\mathrm{PM}}_{2.5}$ ${}^{g}$
LPV-D	48.6	32.6	6.7	12.772	0.2567
MPV-G	47.3	25.97	4.1	0.47	0.126
SPV-G	33.6	9	1.57	0.37	0.117
MNPV-G	34	6.38	3.33	1.24	0.09
HDT-D	50	24.9	6.3	10.2	0.23
MDT-D	24	15	1.5	6.4	0.11
LDT-D	20	12.9	2.9	3.2	0.17
MNT-G	38.4	7.96	1.57	0.37	0.09
PB-D	57.2	33	6.7	12.772	0.35
Taxi-G	74.9	8.7	0.927	0.148	0.117

Notes: ${}^{a}$ In reality, the fuel economy and pollution emission factors may change over time. Due to data availability limitations, we have to make some simple processing and ignore the changes of them in this paper. ${}^{b}$ LPV: large passenger vehicles; MPV: medium passenger vehicles; SPV: small passenger vehicles; MNPV: mini passenger vehicles; HDT: heavy duty trucks; MDT: medium duty trucks; LDT: light duty trucks; MNT: mini trucks; PB: public buses; D: diesel; G: gasoline. ${}^{c}$ Baseline VMT refers to the 2002 national average VMT. For the VMT of LPV-D, MPV-G, SPV-G, MNPV-G, HDT-D, MDT-D, LDT-D and MNT-G, see China Energy Databook v.7.0, October 2008. For the VMT of PB-D and Taxi-G, see [42]. ${}^{d}$ The fuel economy: LPV-D [43]; MPV-G, SPV-G, HDT-D, MDT-D, LDT-D and MNT-G [42]; MNPV-G [44]; PB-D [45]; Taxi-G [46]. ${}^{e}$ The pollution emission factors of CO: LPV-D, PB-D [47]; MPV-G [42], SPV-G, MNPV-G and MNT-G [44]; HDT-D, MDT-D and LDT-D [48]; Taxi-G [46]. ${}^{f}$ The pollution emission factors of ${\mathrm{NO}}_{x}$: LPV-D, PB-D [47]; MPV-G [42]; SPV-G, MNPV-G and MNT-G [44]; HDT-D, MDT-D and LDT-D [48]; Taxi-G [46]. ${}^{g}$ The pollution emission factors of ${\mathrm{PM}}_{2.5}$: LPV-D [49]; MPV-G [50]; SPV-G [51]; HDT-D, MDT-D and LDT-D [48]; PB-D [52]; MNT-G [53]. Due to the unavailable emission factors of ${\mathrm{PM}}_{2.5}$ for Taxi-G and MNPV-G, we assume that they are the same as SPV-G and MNT-G, respectively.

**Table 4:** Elasticities of fuel demand and air pollution emissions from the AIDS model.

Vehicle Types	Fuel Demand Elasticities ${}^{a}$		Pollution Emission Elasticities ${}^{b}$		
	Own-Price	Expenditure	CO	${\mathrm{NO}}_{x}$	${\mathrm{PM}}_{2.5}$
LPV-D	−0.625(0.016)	0.861(0.013)	−0.009	−0.021	−0.010
MPV-G	−1.215(0.003)	0.615(0.006)	−0.046	−0.006	−0.025
SPV-G	−0.940(0.001)	1.133(0.002)	−0.326	−0.097	−0.420
MNPV-G	−0.784(0.014)	0.352(0.042)	−0.116	−0.053	−0.057
HDT-D	−0.898(0.011)	1.313(0.023)	−0.178	−0.300	−0.119
MDT-D	−0.937(0.004)	0.737(0.021)	−0.032	−0.149	−0.042
LDT-D	−0.940(0.006)	1.239(0.017)	−0.134	−0.169	−0.139
MNT-G	−0.796(0.005)	0.872(0.003)	−0.015	−0.004	−0.016
PB-D	−0.459(0.079)	0.286(0.108)	−0.039	−0.090	−0.036
Taxi-G	−0.918(0.001)	0.296(0.012)	−0.031	−0.006	−0.070
			1.025 *	1.026 *	1.031 *

Notes: ${}^{a}$ Values in the parentheses are standard errors. All elasticities are statistically significant at the 5% level. ${}^{b}$ Elasticities with * stand for “expenditure elasticities of pollution emissions”. Other values indicate the “price elasticities of pollution emissions”.

**Table 5:** Elasticities of fuel demand and air pollution emissions from the QUAIDS model.

Vehicle Types	Fuel Demand Elasticities ${}^{a}$		Pollution Emission Elasticities ${}^{b}$		
	Own-Price	Expenditure	CO	${\mathrm{NO}}_{x}$	${\mathrm{PM}}_{2.5}$
LPV-D	−0.482(0.005)	0.233(0.017)	−0.001	−0.001	−0.002
MPV-G	−0.652(0.010)	0.663(0.006)	−0.015	−0.002	−0.008
SPV-G	−1.399(0.019)	1.145(0.003)	−0.385	−0.108	−0.506
MNPV-G	−0.856(0.005)	0.453(0.050)	−0.047	−0.021	−0.023
HDT-D	−0.895(0.063)	1.214(0.015)	−0.125	−0.230	−0.081
MDT-D	−0.587(0.004)	0.708(0.036)	−0.017	−0.081	−0.021
LDT-D	−0.948(0.007)	1.183(0.012)	−0.119	−0.149	−0.123
MNT-G	−0.989(0.024)	0.256(0.023)	−0.005	−0.001	−0.005
PB-D	−0.369(0.059)	0.369(0.095)	−0.011	−0.021	−0.009
Taxi-G	−1.373(0.023)	0.431(0.010)	−0.021	−0.004	−0.047
			0.983 *	0.948 *	0.998 *

Notes: ${}^{a}$ Values in the parentheses are standard errors. All elasticities are statistically significant at the 5% level. ${}^{b}$ Elasticities with * stand for “expenditure elasticities of pollution emissions”. Other values indicate the “price elasticities of pollution emissions”.

**Table 6:** Air pollution emissions and public health losses from the road transport sector ${}^{a}$.

**Panel A: Estimation of Air Pollution Emission Scenarios ${}^{b}$**		CO		${\mathrm{NO}}_{x}$		${\mathrm{PM}}_{2.5}$		**Total ${}^{g}$**
		**Δ Quantity (%) ${}^{c}$**	**Δ Concentration (%) ${}^{d}$**	**Δ Quantity (%) ${}^{c}$**	**Δ Concentration (%) ${}^{d}$**	**Δ Quantity (%) ${}^{c}$**	**Δ Concentration (%) ${}^{d}$**	
**The Results from the AIDS Model: ${}^{i}$**								
Scenario 1 (+30%)		−96.394(−2.777)	−0.008(−0.641)	−17.200(−2.688)	−0.994(−2.116)	−1.133(−2.805)	−0.160(−0.358)	−114.727
Scenario 2 (+5%)		−16.066(−0.463)	−0.001(−0.107)	−2.867(−0.448)	−0.166(−0.353)	−0.189(−0.468)	−0.027(−0.060)	−19.121
Scenario 3 (−40%)		128.525(3.703)	0.011(0.855)	22.933(3.583)	1.326(2.821)	1.511(3.740)	0.213(0.477)	152.969
Scenario 4 (−3%)		9.639(0.278)	0.001(0.064)	1.720(0.269)	0.099(0.212)	0.113(0.280)	0.016(0.036)	11.473
**The Results from the QUAIDS Model: ${}^{j}$**								
Scenario 1 (+30%)		−77.416(−2.230)	−0.007(−0.515)	−11.865(−1.854)	−0.686(−1.459)	−1.000(−2.475)	−0.141(−0.316)	−90.281
Scenario 2 (+5%)		−12.903(−0.372)	−0.001(−0.086)	−1.977(−0.309)	−0.114(−0.243)	−0.167(−0.412)	−0.024(−0.053)	−15.047
Scenario 3 (−40%)		103.222(2.974)	0.009(0.686)	15.820(2.472)	0.915(1.946)	1.333(3.300)	0.188(0.421)	120.375
Scenario 4 (−3%)		7.742(0.223)	0.001(0.051)	1.186(0.185)	0.069(0.146)	0.100(0.248)	0.014(0.032)	9.028
**Panel B: Evaluation of Public Health Losses Scenarios ${}^{b}$**		CO		${\mathrm{NO}}_{x}$		${\mathrm{PM}}_{2.5}$		**Total ${}^{h}$**
		**Δ Premature Deaths ${}^{e}$**	**Δ Losses ${}^{f}$**	**Δ Premature Deaths ${}^{e}$**	**Δ Losses ${}^{f}$**	**Δ Premature Deaths ${}^{e}$**	**Δ Losses ${}^{f}$**	
**The Results from the AIDS Model: ${}^{l}$**								
Scenario 1 (+30%)	linear case ${}^{k}$	−2984	−2553.567	−12515	−10,708.197	−650	−556.189	−16,149(−13,817.953)
	non-linear case ${}^{l}$	−2952	−2525.704	−11,934	−10,210.823	−648	−554.874	−15,534(−13,291.400)
Scenario 2 (+5%)	linear case ${}^{k}$	−497	−425.595	−2086	−1784.699	−108	−92.698	−2692(−2302.992)
	non-linear case ${}^{l}$	−492	−420.907	−1988	−1701.071	−108	−92.477	−2588(−2214.454)
Scenario 3 (−40%)	linear case ${}^{k}$	3979	3404.756	16,686	14,277.596	867	741.585	21,532(18,423.937)
	non-linear case ${}^{l}$	3935	3366.567	15,890	13,596.843	865	739.783	20,690(17,703.193)
Scenario 4 (−3%)	linear case ${}^{k}$	298	255.357	1251	1070.820	65	55.619	1614(1381.795)
	non-linear case ${}^{l}$	295	252.534	1192	1020.466	64	55.485	1552(1328.486)
**The Results from the QUAIDS Model: ${}^{j}$**								
Scenario 1 (+30%)	linear case ${}^{k}$	−2397	−2050.835	−8633	−7386.881	−574	−490.793	−11,604(−9928.509)
	non-linear case ${}^{l}$	−2371	−2028.406	−8231	−7042.598	−572	−489.631	−11,174(−9560.635)
Scenario 2 (+5%)	linear case ${}^{k}$	−399	−341.806	−1439	−1231.147	−96	−81.799	−1934(−1654.751)
	non-linear case ${}^{l}$	−395	−338.039	−1371	−1173.417	−95	−81.603	−1861(−1593.061)
Scenario 3 (−40%)	linear case ${}^{k}$	3196	2734.447	11,511	9849.175	765	654.390	15,472(13,238.012)
	non-linear case ${}^{l}$	3160	2703.872	10,965	9381.761	763	652.802	14,888(12,738.435)
Scenario 4 (−3%)	linear case ${}^{k}$	240	205.084	864	738.688	57	49.079	1161(992.851)
	non-linear case ${}^{l}$	237	202.817	822	703.966	57	48.961	1117(955.746)

Notes: ^a^ We use the 2012 data as the baseline data. ^b^ Scenarios 1 and 2 respectively refer to scenarios for which the prices of gasoline and diesel simultaneously rose by 30% and 5%. Scenarios 3 and 4 respectively refer to prices of gasoline and diesel simultaneously declining by 40% and 3%. ^c^ Δ Quantity is the changes of pollution emissions from road transport (unit: 10,000 tonnes). The values in the parentheses stand for percentage changes. ^d^ Δ Concentration is the changes of air pollutant concentrations (unit: mg/m^3^ for CO, μg/m^3^ for NO_*x*_ and PM_2.5_). The values in the parentheses stand for percentage changes. ^e^ The premature deaths cases caused by acute exposure. ^f^ Unit: millions of RMB yuan. ^g^ The total amount of changes in pollution emissions from the road transport sector (unit: 10,000 tonnes). ^h^ The total number of changes in premature deaths and corresponding economic losses (in the parentheses, million of RMB yuan) caused by air pollution from the road transport sector. In addition, the health damages caused by the joint effects of different air pollutants are very complicated, on which there is no consensus and solid methods to deal with this issue; for simplicity, these effects are ignored in this study. ^i^ The estimation of air pollution emissions (or evaluation of public health losses) is based on the fuel demand system estimated by the AIDS model. ^j^ The estimation of air pollution emissions (or evaluation of public health losses) is based on fuel demand system estimated by the QUAIDS model. ^k^ Results are based on the linear health effect model (see Equation (14)). ^l^ Results are based on the non-linear health effect models (see Equations (15)–(17)).
